# The ordering of expression among a few genes can provide simple cancer biomarkers and signal BRCA1 mutations

**DOI:** 10.1186/1471-2105-10-256

**Published:** 2009-08-20

**Authors:** Xue Lin, Bahman Afsari, Luigi Marchionni, Leslie Cope, Giovanni Parmigiani, Daniel Naiman, Donald Geman

**Affiliations:** 1 Department of Applied Mathematics and Statistics, The Johns Hopkins University, Baltimore, Maryland, USA; 2 Department of Electrical and Computer Engineering, The Johns Hopkins University, Baltimore, Maryland, USA; 3 Department of Oncology, The Johns Hopkins Kimmel Cancer Center, Baltimore, Maryland, USA; 4 Department of Biostatistics, Johns Hopkins Bloomberg School of Public Health, Baltimore, Maryland, USA; 5 Institute for Computational Medicine, The Johns Hopkins University, Baltimore, Maryland, USA

## Abstract

**Background:**

A major challenge in computational biology is to extract knowledge about the genetic nature of disease from high-throughput data. However, an important obstacle to both biological understanding and clinical applications is the "black box" nature of the decision rules provided by most machine learning approaches, which usually involve many genes combined in a highly complex fashion. Achieving biologically relevant results argues for a different strategy. A promising alternative is to base prediction entirely upon the relative expression ordering of a small number of genes.

**Results:**

We present a three-gene version of "relative expression analysis" (*RXA*), a rigorous and systematic comparison with earlier approaches in a variety of cancer studies, a clinically relevant application to predicting germline BRCA1 mutations in breast cancer and a cross-study validation for predicting ER status. In the BRCA1 study, *RXA *yields high accuracy with a simple decision rule: in tumors carrying mutations, the expression of a "reference gene" falls between the expression of two differentially expressed genes, *PPP1CB *and *RNF14*. An analysis of the protein-protein interactions among the triplet of genes and *BRCA*1 suggests that the classifier has a biological foundation.

**Conclusion:**

*RXA *has the potential to identify genomic "marker interactions" with plausible biological interpretation and direct clinical applicability. It provides a general framework for understanding the roles of the genes involved in decision rules, as illustrated for the difficult and clinically relevant problem of identifying *BRCA*1 mutation carriers.

## Background

In principle, an enormous amount of information about biological function and the genetic mechanisms of disease resides in high-throughput data and one of the major challenges of computational biology is to extract this knowledge using techniques from machine learning and statistical inference. In particular, many classification techniques developed in the statistical learning community have been applied to cancer diagnosis, prognosis and sub-type identification based on gene expression microarrays.

Nonetheless, the clinical applications resulting from statistical analyses remain somewhat limited. Indeed, a certain skepticism is well-founded since results, for instance "signatures" and reported error rates, obtained in one study often do not generalize to another. In the case of molecular cancer diagnosis and prognosis from gene expression data, there are several plausible reasons for these difficulties. One issue is certainly the high dimensionality of the data relative to the typical sample size, the well-known "small n, large p" dilemma. A typical microarray data set contains expression values of thousands to tens of thousands of transcripts but for only tens or at most hundreds of samples. This technical barrier can be somewhat lowered by aggregating data from different studies so as to reach samples sizes in the hundreds, but this may still be "small" relative to the complex interactions among the observed variables that one would like to uncover.

Another important obstacle to both biological understanding and clinical applications is the "black box" nature of the decision rules produced by most machine learning classification methods. These rules generally involve a great many genes combined in a highly nonlinear fashion. This is not surprising: by and large, these techniques were developed in other communities, notably pattern recognition, computational vision and computational speech, where data are plentiful and transparency of the decision rules is generally not a criterion for success. In contrast, simplicity and interpretability are highly desirable features for biomedical applications.

Breast cancer prognosis is at the forefront of the application of classification rules based on gene expression, as three such assays have been recently approved for use in clinical management of patients. For a complete review of these assays and their validation see [1]. The three assays differ in several respects: the technology used to measure gene expression, the classification algorithms used, the number of genes considered (2, 21, and about 1900 respectively), the way they were developed, and the degree of their validation on independent populations in real clinical settings. Importantly, none of the classification algorithms used is easily categorized into a well-known machine learning technique. All are based on thresholds applied to compounded continuous scores obtained through a mix of classification techniques, empirical observations, and biological insight applied to the training sets. This puts a barrier between statistical learning and current clinical applications, and emphasizes the need for classification rules that are interpretable and as independent as possible from the specific technology used for the measurement of biological markers, since technology is continuously evolving.

In view of these considerations, achieving statistically stable and biologically relevant results argues for a different strategy, particularly if we wish to go beyond a mere list of "biomarkers" to identifying potential "marker interactions" among several genes. A promising alternative is to base prediction entirely upon the relative expression ordering of a small number of genes. The simplest variation on this theme is to base classification on ratios of expression values, first introduced in a heuristic way in [2] and independently developed as a general, data-driven procedure, the *TSP *algorithm, by [3], and later applied to learn cancer biomarkers and induce elementary prediction rules for cancer diagnosis and prognosis in [4-6]. It has also recently been applied to differentiate between gastrointestinal stromal tumors and leiomyosarcomas [7], resulting in a nearly-perfect two-gene classifier, and to predict response to the farnesyltransferase inhibitor tipifarnib in acute myeloid leukemia [8]. Specifically, one need only compare the expression values among two genes, thus providing a specific hypothesis for follow-up studies.

**Figure 1:**
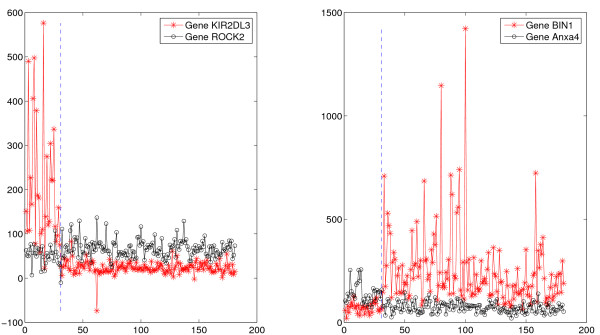
**Expression patterns for two pairs of genes in the Gordon lung study**. In the Gordon lung study, there are two pairs of genes which best discriminate between the MPM and ADCA (lung cancer). The figure shows graphs of the raw expression values for each of the two genes for the 31 MPM and 150 ADCA samples. The decision rule for the left pair is to choose MDM if *KIR2DL3 *is more expressed than *ROCK2 *and choose ADCA otherwise; for the right pair, the rule is to choose MPM if *BIN1 *is less expressed than *Anxa4*.

The *TSP *algorithm is illustrated in Figure 1 for the Lung data from [2], where the objective is to distinguish between malignant pleural mesothelioma (MPM) and adenocarcinoma (ADCA) of the lung. The purpose of this example is only to visualize the *TSP *decision process, not to re-analyze the Lung data. In the left panel, MPM and ADCA samples are well-separated by comparing the expression values of the genes *KIR2DL3 *and *ROCK2 *whereas in the right panel the comparison is based on *BIN1 *and *Anxa4*. The high accuracy obtained, roughly 98%, corroborates the findings in [2], in which several genes are first identified based on fold changes, standard t-tests, expression cutoffs, etc., and then multiple ratios are formed and used both individually and in combination. In contrast, the pairs in Figure 1 are unrestricted, allowing for non-differentially-expressed genes to appear. Whereas ad hoc, and not rank-invariant, the approach in [2] illustrates the power and transparency of simple decision rules.

**Figure 2:**
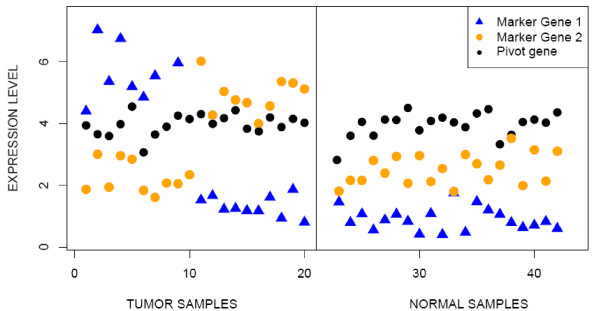
**Phenotype discrimination with gene triples**. Expression pattern of normal and cancer samples, separated by the vertical line. There are two latent subclasses among the cancer samples, represented by the two relative orderings between gene 1 and gene 2. Whereas the normal and diseased samples cannot be separated by this relative ordering alone, refining it by a third gene identifies the normal samples as those for which the pivot gene is the most expressed among the three.

A remaining obstacle to an even broader applicability of the *TSP*   methodology  is the heterogeneity of molecular mechanisms underlying the same disease   phenotype. In cancer, for example, tumors that would look similar under a microscope can present different expression patterns. When this is the case, it is a challenge to identify single pairs with good discrimination. This is illustrated by a simple, artificial example in Figure 2. There are two latent subclasses among the cancer samples, captured by the two relative orderings between gene 1 (in blue) and gene 2 (in orange). These could be two genes whose activity is sufficient to activate the same cancer-related pathway, or they could each flag the activation of alternative cancer-related pathways. Using these two genes alone we cannot distinguish between a normal and an ill patient based on their relative ordering since the cancer phenotype can have either ordering. However, if we combine these two genes with a suitable "reference" gene, the cancer can then be identified, since the reference gene, while relatively stable overall, is the most highly expressed in most of the normal samples but rarely in cancer.

Motivated by these considerations, as well as the broader goal of extending *TSP *to more genes in order to increase accuracy without sacrificing interpretability, we explore the differing roles the genes play in the decision mechanism and apply this methodology to two problems in breast cancer. The first is of direct clinical applicability, while the second is chosen because it provides opportunities for cross-study validation.

The Top-Scoring Triplet (*TST*) classifier is based on the expression orderings among three genes. In both *TSP *and *TST*, "score" refers to the apparent classification rate, defined here as the average of sensitivity and specificity. Given any triplet of genes, the classification rule is then determined by maximum likelihood: choose the class for which the observed ordering is most likely. The probabilities (six for each class) are estimated from the training data. As with *TSP*, there are no parameters to tune and classification results are invariant to any form of data preprocessing and normalization which preserves the ranking among the expression values within a sample. Clearly, *TST *is potentially more discriminating than *TSP *since there are now six possible orderings and this refinement of two-gene orderings can sometimes capture interactions that are not accessible to the *TSP *classifier; in particular, *TST *significantly out-performs *TSP *in detecting BRCA1 mutations. We refer to the family of methods encompassing both *TSP *and *TST *as *RXA*, for "relative expression analysis."

The simplicity of *RXA *for a small number of genes allows for an exploration of the differing roles played by the genes in phenotype distinction. Returning to Figure 1, notice that both genes for the pair on the right are differentially expressed, although *BIN2 *more so than *Anxa4*, whereas clearly only *KIR2DL3 *is differentially expressed for the pair on the left, in which the gene *ROCK2 *serves as a "pivot" or "reference". Moreover, the situation depicted in the artificial example Figure 2 appears in our featured application to detecting BRCA1 mutations:  the top-scoring triplet of genes contains a pivot gene, *TMEM57*, which  always sits between two differentially expressed genes, *PPP1CB* and *RNF14*,  in mutated tumors. *PPP1CB *encodes for a protein shown to directly interact with *BRCA1*, and is expressed at high levels in mutants. *RNF14 *is a co-factor that modulates hormone nuclear receptors activity, including the estrogen and androgen receptors, an activity similar to that of the *BRCA1 *protein itself, and is expressed at low levels in mutants. The role of pivot genes is elaborated in the Discussion.

In summary, we report on i) a computational advance, namely a general *RXA *framework for phenotype identification based on genomic features, including a rigorous and systematic comparison with earlier approaches in a variety of cancer studies; ii) a cross-study validation based on the notoriously hard problem of predicting ER status in breast cancer; and iii) a clinically relevant application to predicting germline BRCA1 mutations in breast cancer, including extensive bioinformatic analysis to provide biological interpretation for the proposed predictor.

## Results

The performance of the *TSP *algorithm has been previously validated in [3-8]. This section is organized as follows. First, general validation results are presented which demonstrate the advantages of bringing in a third gene for a variety of well-known studies in molecular cancer diagnosis and subtype identification from microarray data. Next, we focus on interpreting the decision rules in terms of the different biological roles played by the three participating genes. The main application – detecting BRCA1 mutations – is then presented. Our three-gene classifier achieves an overall accuracy of 94% in cross-validation on the combined van't Veer and Hedenfalk datasets, which well exceeds the performance of several well-known methods. Finally, we present a cross-study validation of our methodology in the context of another important classification problem in breast cancer – predicting ER status.

### General Validation

In Table 1 we compare the classification accuracy of two-gene and three-gene versions of *RXA *for nine cancer datasets summarized in Table 2. The three-gene version is *TST*(10, 10, 10), which restricts all three genes to the ten most differentially expressed; see Methods. In order to ensure a fair comparison, we restricted the two genes in *TSP *to be among the sixteen most differentially expressed. Since the number of ways to select three genes from ten, namely 120, is the same as the number of ways to select two genes from among sixteen, the total number of candidate classifiers is identical.

**Table 1:** Comparison of classification accuracies for a two-gene classifier, *TSP*, and a three-gene classifier, *TST*, for ten cancer studies.

**Data Set**	**Leukemia**	**CNS**	**DLBCL**	**Colon**	**Prostate1**	**Prostate2**	**Prostate3**	**Lung**	**GCM**	**BRCA1**
*TST*	98%	82%	95%	92%	93%	67%	95%	98%	82%	77%
*TSP*	91%	73%	98%	93%	89%	69%	91%	94%	79%	66%

The classification rates are estimated by leave-one-out cross validation. In both cases, the genes are restricted to a subset of differentially expressed genes based on a Wilcoxon rank test; the details appear in §2.3 and §3.1. The subsets are selected to ensure that the number of possible two-gene and three-gene classifiers are the same.

**Table 2:** Nine cancer datasets used for comparing the classification performance of two-gene and three-gene versions of *RXA*.

**Dataset**	**Platform**	**No. of Genes**	**No. of Samples**		**Reference**
			**Class 1**	**Class 2**	
Colon	cDNA	2000	40(T)	22(N)	[40]
Leukemia	Affy	7129	47(ALL)	25(AML)	[41]
CNS	Affy	7129	25(C)	9(D)	[42]
DLBCL	Affy	7129	58(D)	19(F)	[43]
Lung	Affy	12533	31(MPM)	150(ADCA)	[2]
Prostate1	Affy	12600	52(T)	50(N)	[44]
Prostate2	Affy	12625	38(T)	50(N)	[45]
Prostate3	Affy	12626	24(T)	9(N)	[46]
GCM	Affy	16063	190(C)	90(N)	[47]

**Figure 3:**
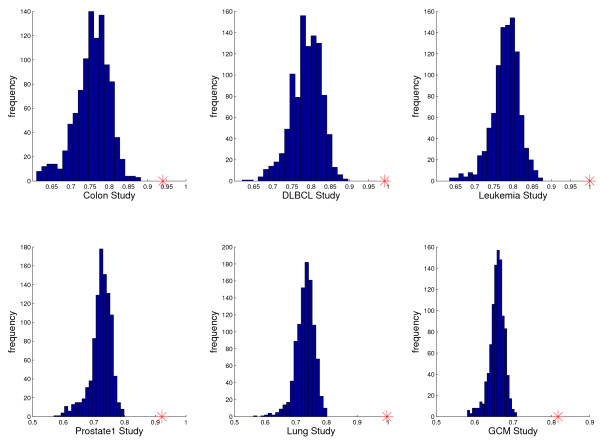
**Significance of top-scoring gene triples in different studies**. The score histograms for the top-scoring gene triple for one thousand random permutations of the class labels. Top row: Colon, DLBCL, Prostate1 data. Bottom row: Leukemia, Lung, GCM data. In each case, the red cross marks the top score on the real data.

Score permutation tests for *TST*(10, 10, 10) for six of the nine datasets are depicted in Figure 3. For each dataset, we randomly permuted the class labels 1000 times and computed the score *S*(*i*, *j*, *k*), the average of sensitivity and specificity, for the top-scoring triplet. Artificial data created in this way preserves both the sample sizes and the overall dependency structure among the genes. Shown is the histogram of scores with the score of the real dataset marked by a red cross. As can be seen, all six scores are highly significant with p-values of zero.

The probability tables for these same six datasets are given in Table 3 and the names of the genes in the top-scoring triple are listed in Table 4. For example, from Table 3 we see that, for the Colon study, the preferred ordering among normal samples is *x*_*j *_<*x*_*i *_<*x*_*k*_, and *x*_*k *_is never the least expressed among these samples; as seen in Table 4, g_*i*_, g_*j*_, g_*k*_, represent *VIP, DARS, FCGR3A*. Similarly, in the Lung data, among the MPM samples, gene *g*_*j *_is always the least expressed, but never so among the cancer samples.

**Table 3:** Relative frequencies of the six possible orderings among the expression values of the top-scoring triple for various studies. .

		**Colon Study**		**DLBCL Study**		**Leukemia Study**	
	**Ordering**	**Tumor**	**Normal**	**DLBCL**	**FL**	**ALL**	**AML**
*π*_1_	*X*_*i *_<*X*_*j *_<*X*_*k*_	0.125	0.045	0.362	0	0.340	0
*π*_2_	*X*_*i *_<*X*_*k *_<*X*_*j*_	0.4	0	0.569	0	0	0.04
*π*_3_	*X*_*j *_<*X*_*i *_<*X*_*k*_	0.075	0.864	0.052	0	0.660	0
*π*_4_	*X*_*j *_<*X*_*k *_<*X*_*i*_	0	0.091	0	0.684	0	0.08
*π*_5_	*X*_*k *_<*X*_*i *_<*X*_*j*_	0.4	0	0.017	0.053	0	0.76
*π*_6_	*X*_*k *_<*X*_*j *_<*X*_*i*_	0	0	0	0.263	0	0.12

		**Prostate1 Study**		**Lung Study**		**GCM Study**	
	**Ordering**	**Cancer**	**Normal**	**MPM**	**ADCA**	**Tumor**	**Normal**

*π*_1_	*X*_*i *_<*X*_*j *_<*X*_*k*_	0.04	0	0	0.12	0.8	0.222
*π*_2_	*X*_*i *_<*X*_*k *_<*X*_*j*_	0.1	0	0	0.827	0.113	0.5
*π*_3_	*X*_*k *_<*X*_*i *_<*X*_*k*_	0.02	0.115	0.258	0.007	0.079	0.022
*π*_4_	*X*_*j *_<*X*_*k *_<*X*_*i*_	0.06	0.807	0.742	0	0	0.044
*π*_5_	*X*_*k *_<*X*_*i *_<*X*_*j*_	0.5	0.019	0	0.02	0.008	0.179
*π*_6_	*X*_*k *_<*X*_*j *_<*X*_*i*_	0.28	0.059	0	0.027	0	0.033

Each portion of the corresponds to one study with two phenotypes. For each study and each phenotype, the table shows the relative frequencies of the six possible orderings among the expression values of the top-scoring triple for the samples in the study. The gene triple (*g*_*i*_, *g*_*j*_, *g*_*k*_) for each study is given in Table 4

**Table 4:** Top scoring triples for each of six studies.

	**Gene name**		
**Study**	**g**_*i*_	**g**_*j*_	**g**_*k*_
Colon	VIP	DARS	FCGR3A
DLBCL	YWHAZ	SNRPB	TXPB151
Leukemia	ZYXIN	CST3	CCND3
Prostate1	HPN	NELL2	TMSL8
Lung	SEPP1	ARL6IP5	BIRC3
GCM	BTN2A1	NT2RAMI	PHLDB2

The three genes in the top-scoring triplet for each of the six studies.

**Figure 4:**
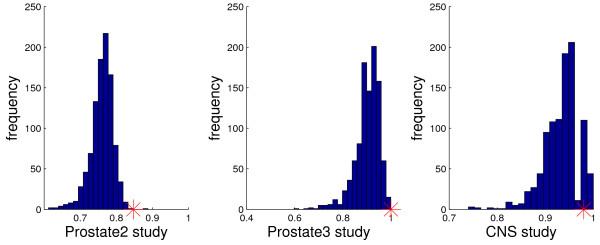
**Insignificance of top-scoring gene triples in some studies**. The score histograms for the top-scoring gene triple for one thousand random permutations of the class labels for Prostate2, Prostate3 and CNS data. Small sample sizes reduce score significance.

The histograms of scores for the top-scoring triple classifier for the other three datasets are given in Figure 4. As can be seen, the top scores for Prostate2 and Prostate3 are still significant, although less so than for the six studies in Figure 3, whereas the top score for the CNS dataset is at best borderline significant. This may not be surprising in view of the very small number of samples for the minority class in both Prostate3 and CNS. Over-fitting is very difficult to avoid with only nine samples for one of the classes; see Table 2.

### Biological Roles

For the *TSP *algorithm, there are two prototypical situations: either both *g*_*i *_and *g*_*j *_are differentially expressed in opposite directions or only *g*_*i *_is differentially expressed and *g*_*j *_serves as a "pivot" or "reference" for *g*_*i*_, in effect a random threshold; see Methods. Call these two cases (*d*, *d*) and (*d*, *p*). A biological mechanism leading to the (*d*, *d*) case may occur when the two genes are involved in competing processes, e.g., one gene may be an oncogene and the other a tumor suppressor gene.

The (*d*, *p*) case was illustrated in Figure 1 for separating malignant pleural mesothelioma (MPM) and adenocarcinomas (ADCA): *ROCK2 *serves as a pivot for *KIR2DL3*, which is up-regulated in MPM samples. The expression of *ROCK2 *is relatively stable across the two phenotypes. The role of "pivot genes" is elaborated in the Discussion section.

**Figure 5:**
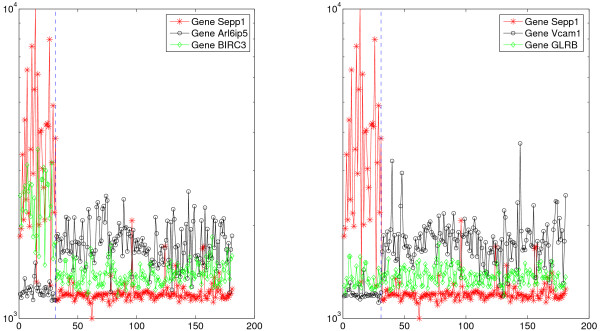
**Expression patterns for two top-scoring triplets for the Lung study**. Left: (d, d, d) case; all three genes are differentially expressed. Right: (d, d, p) case; two of the genes are differentially expressed and the other, GLRB, serves as a pivot.

Several typical cases emerge for three genes. One is (*d*, *d*, *d*), meaning that all three genes in the top-scoring triplet are differentially expressed. This is illustrated for the Lung study in the left panel of Figure 5, where the gene triplet is the one selected by the *TST*(10, 10, 10) algorithm. Another case is (*d*, *d*, *p*), signifying that two of the three genes are differentially expressed and the third is not, serving instead as a reference for the other two. This is illustrated in the right panel of Figure 5; the gene triplet comes from *TST*(10, 10), in which one of the three genes is unrestricted. This is also what emerges for the top-scoring triplet in the BRCA1 study; see again the treatment of pivot genes in the Discussion section

### Identifying BRCA1-related Breast Cancer

We now consider the identification of breast tumors that arose as the result of an inherited deleterious mutation of the *BRCA1 *gene. BRCA1 is a tumor suppressor gene whose altered function is associated with breast, ovarian and other cancers [9]. Deleterious germline mutations of BRCA1 have been estimated to occur in 1 in 40 Ashkenazi Jews and 1 in 400 non-Ashkenazi [10] and are responsible for a significant fraction of inherited breast cancer cases [11]. Genetic testing for germline mutations is available commercially. Testing is expensive and, despite significant recent improvements, still incomplete in its sensitivity [12]. For women newly diagnosed with breast cancer, and a family history of the disease, knowledge of whether they harbor germline mutations is of help in guiding decisions about prevention of contralateral breast cancer and ovarian cancer. Prevention options include radical surgery. Therefore, the availability of inexpensive expression markers for BRCA1 mutations based on tumor samples would significantly improve the clinical management of these women, and contribute to more efficient prevention within their families.

We apply the *TST*(10,10) algorithm (see Methods) to predict BRCA1 mutant status, using two breast cancer gene expression microarray data sets available from the public domain. The raw data can be downloaded from the supporting websites of the two published manuscripts [13,14]. The two studies, designated van't Veer and Hedenfalk, are generated from two different platforms. After data preprocessing and cross-platform matching (as described in Cope, L., Zhong, X., Garrett-Mayer, E., Parmigiani, G. and Gabrielson, E.: Cross-study validation of a molecular profile for BRCA1-linked breast cancers, working paper), we obtain a combined dataset with 1658 features and 118 samples. The two classes (sometimes referred to as "phenotypes" although they refer to properties of germline DNA) are BRCA1-mutant cancers and non-BRCA1 cancers, with sample sizes 25 and 93, respectively.

**Figure 6:**
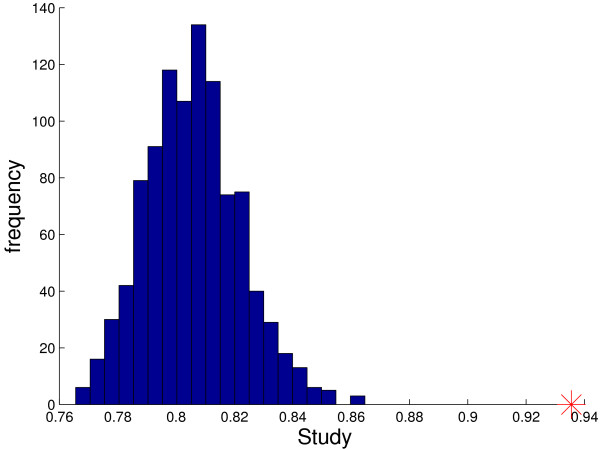
**Significance of the top-scoring triple in the BRCA1 study**. Permutation test for the score of the top-scoring triplet in the BRCA1 study. The score of the top-scoring triplet exceeds the scores under every one of 1,000 random permutations of the class labels, making the triplet found highly significant.

For the *TST*(10,10) algorithm, the score for the top-scoring triplet is .936. The estimated gene expression ordering probabilities for the genes in this triplet are shown in Table 5. Since there are about  × 1658 ≈ 100,000 triplets having at least two differentially expressed genes, a high score might happen by chance. However, a permutation test demonstrates that the p-value of score of the top-scoring triplet is virtually zero; see Figure 6.

**Table 5:** Ordering probabilities for the top-scoring triplet in the BRCA1 study.

**Ordering**	**BRCA1**	**nonBRCA1**
*X*_1 _<*X*_2 _<*X*_3_	0	.204

*X*_1 _<*X*_3 _<*X*_2_	0	.226

*X*_2 _<*X*_1 _<*X*_3_	0	.065

*X*_2 _<*X*_3 _<*X*_1_	0	.172

*X*_3 _<*X*_1 _<*X*_2_	0	.204

*X*_3 _<*X*_2 _<*X*_1_	1	.129

Estimated probabilities for the six possible orderings among the three genes in the top-scoring triplets for each of the two classes. Here, *X*_1_, *X*_2_, and *X*_3 _refer to the expression values of *PPP1CB, TMEM57 and RNF14*, respectively. Mutant samples are characterized by a single preferred ordering whereas non-mutant samples display all orderings.

#### Performance

We compare the performance of the *TST*(10,10) algorithm with *TSP *and four well-known machine learning methods: naive Bayes (NB), *k*-nearest neighbor (*k*-NN), support vector machine (SVM), and random forest (RF). We use the WEKA machine learning package [15] which contains all but *TSP *and *TST*, as well as several R packages. For *TSP *and *TST *we have developed an R package for relative expression analysis (*RXA*) which incorporates all the versions of *TSP *and *TST *used in this paper and which is available for download at . For NB, *k*-NN, SVM and RF, in order to optimize performance, we report the *best *results we obtained by taking either the WEKA default parameters or systematically exploring the parameter spaces, estimating generalization errors with cross-validation. In the case of SVMs, this included trying a wide range of combinations for the scale and penalty parameters with the RBF kernel. Table 6 summarizes the results of LOOCV. As seen, *TST *has the best overall classification accuracy (.936) and the best sensitivity (1.0). (The LOOCV rate for *TST* is the same as the re-substitution rate because the  same top-scoring triple is found in each loop of the cross-validation*.*) Equally noteworthy, *TST *involves only three genes whereas the four traditional machine learning methods use many more.

**Table 6:** Comparison of accuracies in predicting BRCA1 mutations.

**Method**	**TSP**	**TST**	**NB**	**k-NN**	**SVM**	**RF**
Accuracy	.740	.936	.560	.714	.766	.664

Sensitivity	.640	1.00	.280	.600	.640	.360

Specificity	.839	.871	.839	.828	.892	.968

Estimates are based on leave-one-out cross-validation and accuracy is defined as the average of sensitivity, the percentage of correctly classified BRCA1 samples, and specificity, the percentage of correctly classified nonBRCA1 samples.

The top-scoring gene triplet is (*PPP*1*CB, TMEM*57, *RNF*14). Table 5 gives the empirical probability distribution over the six possible orderings of expression values for each of the two phenotypes. Interestingly, the expression values of this triplet are in the same order



for all BRCA1-mutant samples; for nonBRCA1-mutant samples, the probability distribution is dispersed, almost uniform.

In our study there are 12 nonBRCA1-mutant samples which exhibit the relation *RNF*14 <*TMEM*57 <*PPP*1*CB*, of which 7 are "basal-like" tumors, a subtype of breast cancer associated with lack of expression of the estrogen receptor and poor prognosis. Interestingly a strong association between the "basal-like" subtype and BRCA1 mutation has been suggested in a number of molecular and pathological studies [16,17].

**Figure 7:**
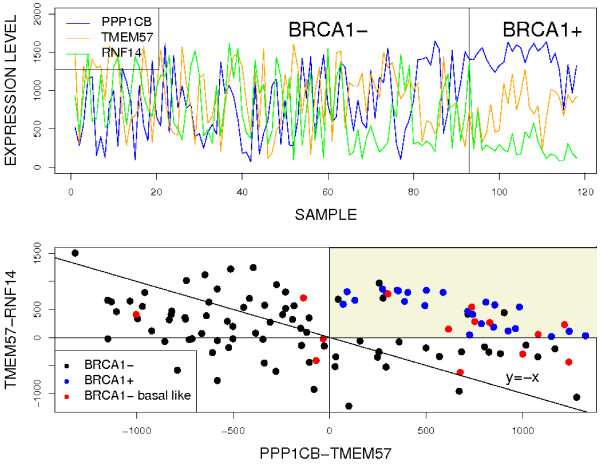
**Expression patterns for the top-scoring BRCA1 triplet**. Top: Expression pattern of the top-scoring triplet (*PPP*1*CB*, *TMEM*57, *RNF*14). The horizontal axis represents samples, with a vertical line separating the *BRCA*1 negative from the *BRCA*1 positive samples. The vertical axis is the *rank *of each genes' expression level within each sample. Bottom: Scatter plot of rank differences. The horizontal axis is the difference between the ranks of *PPP*1*CB *and *TMEM*57 within each sample, while the vertical axis is the difference between the ranks of *TMEM*57 and *RNF*14 for each sample. The shaded area corresponds to the ordering that is unique to the BRCA1 mutant cases.

The top panel in Figure 7 shows the expression pattern of the top-scoring triplet. We can see that *PPP*1*CB *is up-regulated in BRCA1 samples, *RNF*14 is down-regulated in BRCA1 samples, while *TMEM*57 is rather stable across the two phenotypes. Both *PPP*1*CB *and *RNF*14 are highly differentially expressed, actually among the top three, with *p*-values of 2.09*E *- 08 and 7.08*E *- 09, respectively.

The benefit of the reference gene *TMEM*57 is displayed in the bottom panel of Figure 7. The decision boundary of the two-gene *TSP *classifier based on *PPP*1*CB *and *RNF*14 is the line *y *= -*x*: the upper right area corresponds to *PPP1CB *> *RNF14 *and the lower left area corresponds to *RNF14 *> *PPP1CB*. Incorporating *TME*M57 into the decision narrows the upper right "mutant" region to the indicated rectangle where *RNF14 *<*TMEM57 *<*PPP1CB*. The samples in the two triangular areas would be misclassified based on the expression ordering of the gene pair *PPP*1*CB *and *RNF*14 alone whereas they are correctly classified by the *TST *algorithm.

### Cross-Study Validation for ER Status

Estrogen receptor (ER) has been studied in clinical breast cancer for more than 30 years. Approximately two-thirds of all breast cancers are ER+ at the time of diagnosis [18]. The expression of ER has important implications for breast cancer diagnosis and treatment. In particular, ER status correlates well with response to hormonal therapy: ER+ patients are much more likely to benefit from such therapy [18]. Research shows that ER+ and ER- tumors display remarkably different gene-expression patterns [19]. Here we use the *TST *method to classify tumors according to their ER status.

Three breast cancer microarray data sets are included in this study and they are denoted as Miller, Sotiriou and expObreast. The raw data of Miller and Sotiriou can be downloaded from Gene Expression Omnibus or supporting web sites [20,21], and expObreast can be downloaded from [22]. Miller and Sotiriou are generated from the Affymetrix HGU133a microarray platform and expObreast are generated from HGU133plus2. MAS5 was used to pre-process the raw data to get probe sets level data. In order to match the two microarray platforms, we only keep those probe sets that are present on both. There is an 88-patient overlap between Miller and Sotiriou, so we exclude the replicate patients from the original Sotiriou. The sample sizes and the phenotype information are shown in Table 7.

**Table 7:** Phenotype information for the cross-study validation.

**Data Set**	**ER-**	**ER+**	**Total**
Sotiriou	30	65	95

Miller	34	213	247

expObreast	60	113	173

Two types of cross-study validation were performed, each involving learning three classifiers. In the first, there is one classifier for each of the three ways of integrating two of the three microarray data sets to obtain a training set and then using the third one for testing. In the second case, each classifier is trained on only one dataset and tested on the data obtained by merging the other two. We restrict our search to genes on five breast cancer related pathways. There are 69 such genes present on the microarray, which correspond to 128 probe sets. Information about the five pathways and the 128 probe sets can be obtained from the additional files 1 and 2.

Table 8 provides the test results for *TSP *and *TST *(*path*) (see Methods). The results are similar for *TSP *and *TST*, and for the two types of cross-study validation, and both methods generalize well from training to testing. The reason that *TSP *performs as well as *TST *when training on a single dataset is that *TST*, which estimates more parameters, is probably more affected by the reduction in sample size.

**Table 8:** Comparison of methods in prediction of ER status.

**Method**	**Training Data (two data sets)**		
	**Sotiriou+Miller**	**expObreast+Sotiriou**	**expObreast+Miller**
*TSP*	.869 (.950, .788)	.803 (.676, .930)	.727 (.533, .920)

*TST*	.867 (.950, .783)	.876 (.882, .869)	.769 (.600, .938)

	**Training Data (one data set)**		
**Method**	**Sotiriou**	**Miller**	**expObreast**

*TSP*	.883 (.926, .840)	.847 (.789, .904)	.786 (.641, .932)

*TST*	.841 (.936, .745)	.852 (.800, .904)	.806 (.688, .924)

Top: classification results when training on the data set obtained by integrating two of the three microarray data sets and testing on the third one. Bottom: classification results when training on one of the three microarray data sets and testing on the data set obtained by integrating the other two. The numbers in the parentheses are the sensitivity and specificity levels respectively.

**Figure 8:**
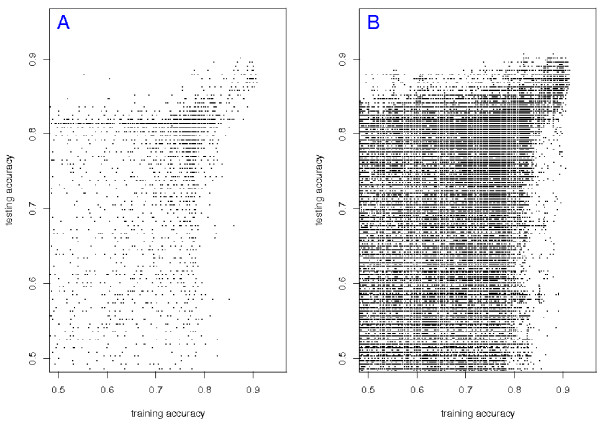
**Reproducibility of top-scoring pairs and triples**. Panel A: Each point represents a candidate gene pair. For each pair, the probabilities of the two orderings are estimated on the combined expObreast and Miller datasets and the classifier is tested on Sotiriou. The *x*-coordinate of a point represents the training accuracy and the *y*-coordinate represents the testing accuracy. Panel B: Same as left panel, but for all triples of genes.

Figure 8 shows the reproducibility of top-scoring pairs and triplets. In both pictures there is an antenna located at the upper right corner. These are the pairs (triplets) that work well on both training and testing data sets, and hence we expect it to involve a small minority of points. These two pictures indicate that both *TSP *and *TST *are reproducible across studies.

## Discussion

### Function and Interactors of *TST *Genes

In addition to the role of *BRCA*1 mutations in familial breast cancer, the reduced expression, or incorrect sub-cellular localization of *BRCA*1 protein, are postulated to be important also in the pathogenesis of sporadic breast cancer. Evaluation of *BRCA*1 protein expression in 1940 breast cancer cases has shown that about 50% of the tumors showed loss of nuclear expression or cytoplasmic localization of the protein [23]. The evidence of biological roles of *BRCA*1 reveals multiple functions for this protein, that may contribute to its tumor suppressor activity, including regulation of cell cycle progression, DNA repair, DNA damage-responsive cell cycle check-points, apoptosis, and the regulation of a set of specific transcriptional pathways like the androgen receptor's and *ESR*1 (reviewed in [24]).

The top scoring triplet identified in our *BRCA*1 analysis involves *PPP*1*CB*, *TMEM*57, and *RNF*14. The *PPP*1*CB *gene is located on chromosomal cytoband 2p23, and encodes one of the three catalytic sub-units of the phosphatase-1 (PP1) serine/threonine-specific protein phosphatases. This multimeric complex is involved in protein dephosphorylation within a variety of cellular processes, including cell division, muscle contractility, glycogen metabolism, protein synthesis, and HIV-1 viral transcription.

The *RNF*14 gene, also known as *ARA*54, is located on chromosomal cytoband 5q23.3-q31.1 and encodes for a protein involved in the modulation, by direct protein-protein interaction, of a number of hormone nuclear receptors, including the androgen receptor (*AR*), the estrogen receptor (*ESR*1), and the growth hormone receptor (*NR*3*C*1). BRCA1 has been shown to directly interact with the andogen receptor, increasing androgen-dependant transcription [25,26]. This supports our observation that the AR cofactor, *RNF*14 has very low expression in those samples in which BRCA1 function is impaired. Finally, the *TMEM*57 gene, located on chromosomal band 1p36.11, encodes for a protein developmentally regulated in the brain, with predominant expression in differentiating neurons [27]. The biological role of this protein is not yet known.

**Figure 9:**
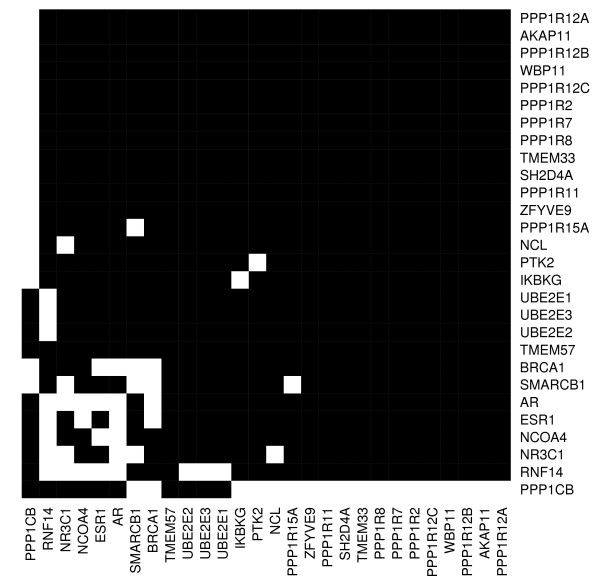
**Protein-protein interaction adjacency matrix**. Protein-protein interaction adjacency matrix for all the protein-protein interactors of *PPP *1*CB*, *RNF*14, *TMEM*57. Protein names appear as columns and row names, direct interaction are shown in white.

Since a recent study by Winter and colleagues showed that all three isoforms of PP1, including *PPP*1*CB*, interact with BRCA1 [28] we used protein-protein interaction (PPI) data to understand whether the triplet genes (*PPP*1*CB*, *TMEM*57, and *RN F *14) directly interact with each other and with BRCA1. In addition to the interactions described by Winter and colleagues, PPI interaction gene lists included information from three sources: the Human Protein Reference Databases (HPRD) [29], BioGrid [30] and the Biomolecular Interaction Network Database (BIND) [31]. All protein-protein interactions reported in any of these databases to involve the triplet genes were used, regardless of the techniques employed to identify the interaction, or the type of evidence supporting its inclusion in the databases. We represented PPI data as adjacency matrices (see Figure 9) and displayed the corresponding networks using undirected graphs, where each node represents a protein while edges represent interactions.

**Figure 10:**
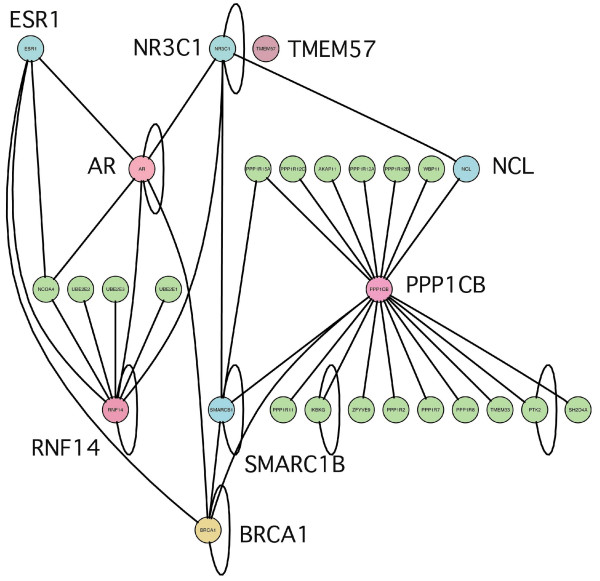
**Protein-protein interaction network**. Protein-protein interaction network involving *PPP*1*CB*, *RNF*14, *TMEM*57, and *BRCA*1. *TMEM*57 has no interactors, while *RNF*14, *TMEM*57 are connected through *BRCA*1 and *AR*, *ESR*1 and *BRCA*1, through *SMARC*1*B *and *NCL*, or through *NR*3*C*1 and *SMARCB*1. Genes from the top-scoring triplets are shown in pink, *BRCA*1 in yellow, the other connecting proteins in light blue.

No PPI interactions were reported for the pivot gene *TMEM*57, while the other two genes (*PPP*1*CB*, *RN F *14) were found to interact with several other proteins. Although none of such inter-actors is in common between the two proteins, the PPI network revealed a number of shortest paths connecting these two proteins and involving a minimum of two proteins (Figure 10). One such path accounts for *BRCA*1 and *AR*, while the other involves *ESR*1, *SM ARCB*1 (SWI/SNF related, matrix associated, actin dependent regulator of chromatin, subfamily b, member 1), *NR*3*C*1 (the growth hormone nuclear receptor), and Nucleolin (*NCL*). Overall, this analysis suggests that *RN F *14 and *PPP*1*CB *are part of a regulatory network modulating gene expression and involving *BRCA*1 and a number of hormone nuclear receptors.

### Pivot Genes

As has been seen, one very interesting scenario that often arises in the application of *TSP *and especially in the application of *TST*, is that one of the genes appears to play a passive role, serving as a pivot point or benchmark expression level for the others. This situation is illustrated well in Figure 2, where the key biological signal of cancer is that at least one of the pathway-activator genes is expressed above the level of the pivot gene. It is difficult to assign roles to the genes in a triplet observed in experimental data with any certainty, but expression patterns consistent with the pivot gene theory have been observed very frequently in a wide variety of experiments and we think it important to consider the implications of this when developing and evaluating methodology. In *Finding Triplets in Practice *of the Methods section, we present practical methods for reducing the triplet search space by recognizing such a division of labor among the genes in a triplet and pre-filtering for genes that, say, could serve as a pivot. In this section we discuss additional biological and technical consequences of the pivot gene theory.

There are technical issues associated with expression microarrays that make it difficult to find a good pivot gene in practice. The intensity level of a microarray probe depends on a variety of technical variables in addition to the biological variable of interest, transcript abundance, and so the measured intensity for gene A may exceed that of gene B even when B is present in greater quantity. In single color microarrays, these probe effects can overwhelm sample to sample differences in gene expression, driving correlations in excess of 95% when expression data obtained from very different samples, but measured on the same array platform are compared. This works two ways: the availability of a large number of probes with relatively constant intensities at various levels should make it quite easy to find efficient pivots when working on a single platform, while on the other hand, the selected pivot gene may not sit at the same level relative to its partners in the triplet when measured by another technology.

Two color arrays offer additional technical challenges and introduce study design issues as well. Classical house-keeping genes, expressed at near constant levels in all cells, should yield expression ratios of 1 for any two samples and so may not work well as benchmark expression levels for other genes. The use of two different dyes for the two samples on an array introduces a technical effect that continues to slightly bias the estimated ratios of individual genes even though the broadest effects are well controlled by standard pre-processing methods [32-34]. Thus, as on single color arrays, pivot genes identified on a two-color platform may be effective only within that technology.

An additional concern arising in two-color studies is the fact that both samples on an array contribute to the expression ratio. The reference samples included in one study may determine the level of a potential pivot gene or in extreme cases, even drive apparent differential expression that is in fact not present in the population of interest and which therefore will not be observed in another study with a different design.

The successes of the *RXA *approach clearly demonstrate that these technical challenges can be overcome, and we believe that steps can be taken in implementation to minimize the threat to performance. Careful preprocessing to minimize the influence of technical effects is a crucial step. Principled pre-filtration of array features, as discussed in *Finding Triplets in Practice *of the Methods section, could help by eliminating a large number of apparently irrelevant and possibly misleading probes from consideration. We also recommend that the *RXA *classifiers be made as robust as possible by maximizing the diversity of samples and platforms included in the training set.

### Biological Findings

In the present study we apply the *TST *algorithm to predict BRCA1 mutant status using data from the public domain. Within the training set, our approach enables a correct classification of all BRCA1 mutants considered, while only 12 sporadic breast cancers are misclassified. A number of factors may account for the BRCA1-like behavior of some of the wildtype tumors. For example, BRCA1 function may be altered by somatic mutation, methylation or other genetic alterations, or it may be impaired by incorrect cellular localization. The "basal-like" subtype of breast cancer is associated with lack of expression of the estrogen receptor and poor prognosis. A strong connection between the "basal-like" subtype and BRCA1 loss of function has been suggested in a number of molecular and pathological studies [16,17]. Among the 12 sporadic breast cancer that were misclassified by *TST*, 7 are "basal-like". Since there are 14 "basal-like" samples in total, the probability of seeing 7 of them in 12 randomly chosen samples is indeed small (.0002). Therefore, the "basal-like" types are over-represented in the 12 nonBRCA1-mutant samples which are classified as BRCA1-mutant cancers by *TST*, arguing for the biological relevance of the top-scoring triplet.

The analysis of the protein-protein interactions among the top-scoring triplet genes and *BRCA*1 further suggests that the classifier has a biological foundation. No interactors were found for *TMEM*57, possibly reflecting its role in the classifier as the pivot gene, while the other two genes showed interactions with many other proteins   involved in breast cancer biology including the *BRCA1 *protein itself.

*PPP*1*CB *is one of the three catalytic sub-units of the protein phosphatase PP1. A 2007 study shows that all three isoforms of PP1 interact with BRCA1 [28]. In the same study, RT-PCR expression analysis indicates that sporadic breast cancers have lower expression levels of *PPP*1*CB *than do normal tissues. The gene expression microarray data that we use show a higher expression level of *PPP*1*CB *in BRCA1-mutant cancers than in sporadic breast cancers. Moreover, the literature also suggests that *PPP*1*CB *is a discriminator gene between BRCA1-mutant and BRCA2-mutant tumors for both breast and ovarian cancers [35].

Although no direct links exist between RNF14 and BRCA1, our PPI analysis shows that indirect interactions occur, and they involve important proteins in breast cancer biology, like the estrogen and androgen receptors. The expression of the estrogen receptor is a fundamental prognostic factor in breast cancer: hormonal therapy is used in adjuvant settings based on whether cancer cells express it or not. Androgen receptor signaling is also emerging as a relevant pathway for breast cancer biology. Unlike prostate cancer, where *AR *is sustaining growth of cancer cells, androgen signaling in breast cancer represents a restraint to cancer cell growth, and it has been shown that *AR *expression correlates with a better prognosis [36]. Interestingly, reduced or altered *BRCA*1 protein expression has been shown to be associated with lack of progesterone and *ESR*1, and expression of the *AR *[23]. From this perspective, it is important that *AR *activity is modulated by numerous factors, including *RNF*14 and *BRCA*1 [36].

While elucidation of the exact mechanisms by which loss of *BRCA*1 function affects expression of *PPP*1*CB *and *RNF*14 awaits additional laboratory work, the evidence we presented strongly suggests that the *TST *classifier described here has properties that go beyond classification performance and that are capturing genuine biological mechanisms underlying breast cancer pathogenesis.

## Conclusion

We have developed and validated a general *RXA *approach to building simple and interpretable classifiers using trios of features. Other approaches have been advanced for selecting informative gene triplets and three-gene interactions from expression microarray data. Recently, methods based on fuzzy logic [37], liquid association [38] and a three-way interaction model [39] have been proposed. In [37], activator-repressor-target triplets are identified using logical relationships among the genes. Liquid association is aimed at capturing the dynamic association between a pair of genes; the correlation between the expression values of a gene pair depends on the expression level of a third gene. The three-way interaction model is similar, except the third gene plays the role of a qualitative switch rather than a continuous measure as in liquid association. However, none of these approaches involve inferring phenotype-specific models or classifiers, and none are rank-based.

While statistical and machine learning techniques have contributed  significantly to the interpretation of the large and complex data sets  generated by high throughput genomic techniques, the direct application of these techniques in the clinical management of patients is slowed by challenges in interpretability and cross-study reproducibility. Algorithms based on the relative level of a small number of genomic features provide a formidable simplification, yielding progress in both interpretability and reproducibility, often at little or no cost in terms of accuracy. This article demonstrates a new incarnation of this philosophy, based on three-gene classifiers, provides a general framework for understanding the roles of the genes involved, and illustrates its potential in the difficult and clinically relevant problem of identifying *BRCA*1 mutation carriers.

## Methods

Let **X **= (*X*_1_, *X*_2_,..., *X*_*G*_) denote the expression values of *G *genes (*g*_1_, *g*_2_,..., *g*_*G*_) on an expression microarray. We regard **X **as a random variable. Our objective is to use **X **to distinguish between two conditions or phenotypes, denoted *Y *= 1 and *Y *= 2, for example "BRCA1 mutation" vs "no BRCA1 mutation", "ER+" vs "ER-" status, or "tumor" vs "normal". The class label *Y *is another random variable.

A classifier *f *associates a class label *f*(**X**) ∈ {1, 2} with each expression vector *X*. It is learned from a training set ℒ with *N *independent and identically distributed samples of (**X**, *Y*), among which there are *N*_1 _samples of class 1 and *N*_2 _= *N *- *N*_1 _samples of class 2. In order to evaluate the performance of *f*, we estimate the generalization error *e*(*f*) = *P*(*f*(**X**) ≠ *Y*) using either an independent test set (in the ER status study) or cross-validation (in the BRCA1 study). The classification rate is 1 - *e*(*f*). In the absence of specific prior information about class likelihoods, and in order to balance sensitivity and specificity, we assume *P*(*Y *= 1) = *P*(*Y *= 2) = 0.5; this makes more sense than using the frequencies  and  observed in the training samples as these can be somewhat arbitrary and usually do not reflect the population statistics. Equivalently, we measure performance by the average of *sensitivity*, defined by *P*(*f*(**X**) = 1|*Y *= 1) and *specificity*, defined by *P*(*f*(**X**) = 2|*Y *= 2).

Given any set of *n *genes {*g*_*i*_, *g*_*j*_,...}, there are *n*! possible orderings among the corresponding expression values {*X*_*i*_, *X*_*j*_,...}. Our decision rules are based only on the ordering or ranks of the expression values within a sample. For *n *= 2, there are clearly two possible orderings: *X*_*i *_<*X*_*j *_and *X*_*i *_≥ *X*_*j*_. For *n *= 3 there are six possible orderings among {*X*_*i*_, *X*_*j*_, *X*_*k*_}. (Ties are very rare, but there is a simple mechanism for handling them that we explain below.)

### Brief Review of TSP

In [3] and subsequent papers about *TSP*, the discriminating power of each pair of genes (*g*_*i*_, *g*_*j*_) was measured by the "score" |*P*(*X*_*i *_<*X*_*j*_|*Y *= 1) - *P*(*X*_*i *_<*X*_*j*_|*Y *= 2)|, where the two probabilities are estimated from the training samples. Moreover, in [5] a "secondary score" was introduced which allows for a *unique *top-scoring pair to be selected in case several pairs of genes obtained the same primary score.

Suppose (*g*_*i*_, *g*_*j*_) is the top-scoring pair of genes and assume these genes are ordered so that *P*(*X*_*i *_<*X*_*j*_|*Y *= 1) > *P*(*X*_*i *_<*X*_*j*_|*Y *= 2). The TSP classifier *f*(**X**) depends only on the observed ordering between *X*_*i *_and *X*_*j*_, and chooses the class for which this ordering is the most likely:



Notice that the average of sensitivity and specificity of *f *is



Hence, maximizing the difference of probabilities over all pairs (*i*, *j*) is the same as maximizing the average of sensitivity and specificity, and hence consistent with our measurement of performance.

### TST: Gene Triplets

Now consider any gene triplet {*g*_*i*_, *g*_*j*_, *g*_*k*_}; the six possible orderings will be denoted by *π*_1_,..., *π*_6_; see the lefthand panel of Table 3. Again, for simplicity, we've assumed no ties.

For each possible ordering *π*_*m*_, *m *= 1,..., 6, let *p*_1_,..., *p*_6 _(resp., *q*_1_,..., *q*_6_) be the probabilities of the corresponding events under *Y *= 1 (resp., *Y *= 2). For instance, *p*_2 _= *P*(*X*_*i *_<*X*_*k *_<*X*_*j*_|*Y *= 1) and *q*_3 _= *P*(*X*_*j *_<*X*_*i *_<*X*_*k*_|*Y *= 2). These probabilities are estimated from the relative frequencies in the training set. (Ties are handled in a natural way: instead of incrementing the count of a single permutation by one, the count of every possible one of the tie-breaking permutations is incremented by the reciprocal of the number of such permutations. For example, if *X*_*i *_= *X*_*j *_<*X*_*k *_for some sample, the counts of permutations (*i*, *j*, *k*) and (*j*, *i*, *k*) are each incremented by 1*/*2.) These relative frequencies are displayed in Table 3 for six different studies. For example, for the Colon study, 40% of the samples exhibit the ordering *x*_*i *_<*x*_*k *_<*x*_*j *_for the top-scoring triple.

Given any gene triple, the associated classifier *f*_*ijk*_(**x**) depends only on the ordering among *x*_*i*_, *x*_*j*_*, x*_*k *_and chooses the class for which the ordering is most likely. That is, if the ordering *π*_*m *_is observed among *x*_*i*_, *x*_*j*_*, x*_*k*_, then



(If *p*_*m *_= *q*_*m*_, the decision is split between the two classes.) Again, the score of the triple is just the average sensitivity and specificity of *f*_*ijk*_, which can be expressed in terms of {*p*_*m*_} and {*q*_*m*_}:



For example, in the Leukemia study, the triple in Table 3 has a perfect score: *S *= 1.

If multiple gene triplets achieve the same top score, a secondary score is used to break the tie and select a unique top-scoring triplet. For any triplet (*g*_*i*_, *g*_*j*_, *g*_*k*_), the secondary score is the sum of the three pair scores Γ(*i*, *j*, *k*) = *S*(*i*, *j*)+*S*(*i*, *k*)+*S*(*k*, *j*).

### Finding Triplets in Practice

As the examples in Table 1 and Figure 2 show, adding a third gene to a gene pair may improve performance. But it also raises computational and estimation issues. While the complexity of an unrestricted search is evidently order *G*^2 ^for *TSP*, it is order *G*^3 ^for *TST*. With thousands of transcripts, it is not feasible to score all possible triplets. A more serious concern, given the sample sizes, is over-fitting.

To address both of these issues, we consider three methods for accelerating the search and preventing over-fitting, all based on filtering the full set of *G *genes. Two are based on standard gene filtering with statistical tests of significance and the third is based on utilizing prior biological information. Undoubtedly some information can be lost; in fact, there are examples of studies in which the (cross-validated) performance of unrestricted *TSP *exceeds that of a search confined to differentially expressed genes. However, the results reported here show that, for *TST*, the amount of remaining information is sufficient for high-accuracy classification.

There are several different ways to measure differential expression; we use the Wilcoxon rank sum test. In keeping with the overall rank-based nature of *RXA*, we do not calculate test statistics based on raw expression values. Instead, we first replace the expression value of each gene by its rank within the sample. The gene with the smallest expression value has rank 1, the next smallest rank 2, and so forth up to rank *G*. The expression data from the *n*-th sample becomes (*R*_1*n*_, *R*_2*n*_,..., *R*_*Gn*_) where *R*_*in *_is the rank of gene *g*_*i *_within the sample. We then assign a p-value to each gene *g*_*j *_based on the Wilcoxon rank sum test for the two samples  and .

**• Three Differentially Expressed Genes**: The *TST*(10, 10, 10) algorithm restricts the search for triplets to the ten most differentially expressed genes in the dataset. For *G *≈ 10^4^, this reduces the search space from order 10^12 ^to  = 120, in which case finding the top-scoring triple, even estimating error rates with cross-validation, is very fast. Equally importantly, the problem of over-fitting – find spurious triples – is virtually eliminated, as permutation tests demonstrate; see *General Validation *in the Results section. Of course the disadvantage is that pivots are excluded.

**• Two Differentially Expressed Genes**: The *TST*(10, 10) algorithm restricts two of the three elements of the triplet to the ten most differentially expressed genes; the third gene may be chosen from among all genes in the study. This allows for pivots but is still manageable computationally.

**• Restrictions to Appropriate Pathways**: The last option, denoted *TST*(*path*), restricts all three genes to lie in certain pathways related to the phenotypes. This is based on the assumption that genes on related pathways behave differently from one phenotype to the other, and thus their ordering relationship may change accordingly. Using appropriate prior information, we then reduce the search space and concentrate on biological meaningful gene sets.

In view of the "reversal" nature of the *TSP *and *TST *decision rules, another possibility, not pursued here, would be to initially search for pairs of genes which are *negatively *correlated.

### Estimating Classification Rates

Once a gene pair (*TSP*) or gene triplet (*TST*) is chosen, classification is based on maximum likelihood for the observed ordering. If an independent test study is available, we use these samples to estimate prediction accuracy. Otherwise, we use leave-one-out cross validation (LOOCV): one sample is left out from the training data, the top-scoring gene triplet is selected form the remaining data and the corresponding classifier is applied to the left-out sample. The estimated prediction rate is then , where *e*_*i *_and *e*_2 _are the total numbers of misclassified samples for classes 1 and 2 respectively. Naturally, filtering is performed within each loop and the top-scoring triples may vary from loop to loop. The genes reported are those which are found on the whole training set.

## Authors' contributions

DG and DN conceived the methodology, and LC, DG, XL, DN and GP designed the study. LC, DG, XL and LM wrote significant portions of the manuscript. BA and XL carried out the bioinformatics and statistical analysis of the microarray data, and LM and GP performed the biological analysis. DN, XL and BA developed the R package for RXA. All authors have critically reviewed and approved the manuscript.

## Supplementary Material

Additional file 1 **Five breast cancer related pathways**. A list of the genes appearing in each of five pathways related to breast cancer. Click here for file

Additional file 2 **Probe sets on the pathways**. The probe identifiers for the genes listed in the five breast cancer related pathways Click here for file
